# Still Excited, but Less Aroused—The Effects of Nutritional Ketosis on Epinephrine Response and Hypothalamic Orexin Neuron Activation Following Recurrent Hypoglycemia in Diabetic Rats

**DOI:** 10.3390/metabo13010042

**Published:** 2022-12-27

**Authors:** Polina E. Nedoboy, Melissa M.-J. Farnham

**Affiliations:** Heart Research Institute, The University of Sydney, Newtown, NSW 2042, Australia

**Keywords:** counterregulatory response, epinephrine, insulin-induced hypoglycemia, ketosis, ketogenic diet, STZ-diabetes, rat model, orexin neurons

## Abstract

Hypoglycemia-associated autonomic failure (HAAF) is a serious, life-threatening complication of intensive insulin therapy, particularly in people with type 1 diabetes. The ketogenic diet is reported to beneficially affect glycemic control in people with type 1 diabetes, however its effects on the neurohormonal counterregulatory response to recurrent hypoglycemia and HAAF development are understudied. In this study we used Sprague Dawley rats to establish a HAAF model under non-diabetic and streptozotocin (STZ)-induced diabetic conditions and determined how nutritional ketosis affected the neurohormonal counterregulation and the activity of energy-sensing orexin (OX) neurons. We found that antecedent hypoglycemia diminished the sympathoexcitatory epinephrine response to subsequent hypoglycemia in chow-fed non-diabetic rats, but this did not occur in STZ-diabetic animals. In all cases a ketogenic diet preserved the epinephrine response. Contrary to expectations, STZ-diabetic keto-fed rats showed reduced OX activity in the recurrent hypoglycemia group, which did not occur in any other group. It is possible that the reduced activation of OX neurons is an adaptation aimed at energy conservation accompanied by diminished arousal and exploratory behaviour. Our data suggests that while a ketogenic diet has beneficial effects on glycemia, and epinephrine response, the reduced activation of OX neurons could be detrimental and warrants further investigation.

## 1. Introduction

Historically, ketogenic diets were used successfully as dietary treatment options for drug-resistant epilepsy [1], and glycemic management of type 1 diabetes mellitus, before the discovery of insulin [2,3]. In recent years, ketogenic diets re-emerged as popular weight-loss regimens in the general population, with some evidence of positive effects on glycemia, HbA1c and blood lipid profile in people with type 2 diabetes [4]. There are mounting case reports and small-scale studies describing the benefits of the ketogenic diet in patients with type 1 diabetes, particularly related to improvements in glycemic control and variability, and reduction in insulin requirements [5,6,7]. However, there are potential risks associated with ketogenic diet consumption by type 1 diabetes patients, such as incidents of severe hypoglycemia and ketoacidosis, which are reported [7,8], but remain less investigated. Given the recent increase in ketogenic diet adherence in people with type 1 diabetes, and a non-trivial rise in new-onset type 1 diabetes diagnoses during the global COVID-19 pandemic [9,10,11], it is important to study the effects of diet-induced ketosis, and potential diabetes-related complications in the context of type 1 diabetes.

Hypoglycemia is an unfortunate side-effect of intensive insulin therapy and can occur when an inappropriately large dose of insulin is injected. Normally, people with type 1 diabetes quickly become aware of hypoglycemia symptoms and take measures to correct hypoglycemia by ingesting carbohydrates. These symptoms and responses are realised through a cascade of highly coordinated neurohormonal counterregulatory reflex responses orchestrated mostly by the brain due to its high and nearly obligatory requirements for glucose as energy source [12]. Repeated episodes of hypoglycemia can lead to impaired awareness of hypoglycemia, a clinical manifestation of hypoglycemia-associated autonomic failure (HAAF), which is reported in up to 40% of people with type 1 diabetes of long duration [13]. Multiple mechanisms are proposed to play a role in the development of HAAF [14], which is a dangerous condition where the sympathoadrenal counterregulatory response to hypoglycemia is suppressed and delayed [15]. HAAF leaves patients unable to both physically sense, and behaviourally respond, to an episode of hypoglycemia, putting them at increased risk of future severe hypoglycemias, seizures, coma, and death. Currently, there is no treatment for HAAF; patients must simply attempt to avoid hypoglycemic episodes by carefully managing their insulin therapy.

Ketone bodies, either produced endogenously during fasting or through the consumption of a ketogenic diet, or provided exogenously in the form of a supplement, provide the brain with an additional energy source during hypoglycemia [16,17], thereby sustaining neuronal function for longer [18]. Somewhat surprisingly, the symptoms of hypoglycemia are reported to be delayed or completely absent in non-diabetic individuals with elevated blood ketones [19]. However, studies in non-diabetic rats with diet-induced ketosis demonstrated a delayed, but preserved epinephrine counterregulatory response to a single insulin-induced hypoglycemia [17], suggesting that although elevated ketones do not improve the loss of hypoglycemia symptoms, the reflex sympathoadrenal response, responsible for restoring blood glucose levels, remains functional. In contrast, the glucagon counterregulatory response to neuroglycopenia was attenuated in mice fed a ketogenic diet for 21 days [20], suggesting a differential effect of nutritional ketosis on the components of hypoglycemia counterregulatory response. At present, there is paucity of published pre-clinical or human data describing the effects of nutritional ketosis on neuroendocrine and metabolic responses to recurrent hypoglycemia in either non-diabetic or diabetic conditions. 

In the current study, we established a rat model of HAAF in non-diabetic and STZ-diabetic animals and investigated the effects of diet-induced ketosis on baseline physiological parameters and neurohormonal responses to recurrent hypoglycemia. We found that in healthy rats the initial blood glucose concentrations inversely correlated with hypoglycemia-stimulated plasma epinephrine. The opposite was true for baseline blood ketones and epinephrine. We showed that the epinephrine counterregulatory response to recurrent hypoglycemia is preserved in ketogenic diet-fed STZ-diabetic rats not supplemented with insulin. The activity of low glucose-sensing hypothalamic orexin/hypocretin (OX) neurons, however, was significantly reduced by the repeated hypoglycemia in this group of animals.

## 2. Materials and Methods

### 2.1. Animals 

Animal work was approved by the Sydney Local Health District Animal Welfare Committee (AWC #2018/014) and conducted according to the Australian Code of Practice for the Care and Use of Animals for Scientific Purposes, New South Wales Animal Research Act 1985. Adult male Sprague Dawley rats (7 weeks old) were group housed (3 rats per cage) at the Heart Research Institute biological facility at 23–25 °C, 50–60% humidity, 12 h dark/light cycles.

### 2.2. Streptozotocin (STZ)-Induced Diabetes

After a one week acclimatization period, rats were randomly assigned into non-diabetic (Non-D) and streptozotocin diabetic (STZ-D) groups. Diabetes was induced by a single intraperitoneal injection of STZ (Streptozocin, S0130, Sigma-Aldrich, Macquarie Park, NSW, Australia, 60 mg/kg, dissolved in citrate buffer, pH 4.5) to overnight fasted animals under light isoflurane anaesthesia. Blood glucose (BG) was measured 48 h after STZ injection and all animals with BG ≥ 16 mmol/L were deemed diabetic (18.7–35.8 mmol/L range). Non-diabetic controls were injected with citrate buffer. One week later, rats were further divided into four experimental groups: chow fed (Standard rodent chow, 4.6% fat, 19% protein, 59.9% total carbohydrate, Specialty Feeds, Glen Forrest, WA, Australia)—non-diabetic (Non-D Chow) and STZ-diabetic (STZ-D Chow); ketogenic diet fed (SF10-053 Ketogenic Rodent Diet, 69% fat, 16% protein, 1.2% digestible carbohydrate, Specialty Feeds, Glen Forrest, WA, Australia)—non-diabetic (Non-D Keto) and STZ-diabetic (STZ-D Keto). STZ-D Chow animals were implanted subcutaneously with 1–2 sustained-release insulin pellets (RES-14PC Linplant, Linshin, Canada) according to manufacturer’s instructions. One week after the insertion of insulin implants and confirmation of BG reduction, the dietary interventions were initiated and maintained for 3 weeks.

### 2.3. Hypoglycemia-Associated Autonomic Failure (HAAF) Protocol

Following 3 weeks of either Chow or Keto diet, baseline BG and β-hydroxybutyrate (BHB) were measured with a handheld glucometer and corresponding strips (LifeSmart 3-in-1 Multifunctional Monitoring System with Glucose and Haematocrit Strips for BG measurements, Genesis Biotech Pty Ltd., QLD, Australia; and Abbott Optium Neo glucose and ketone meter with Freestyle Optium ketone strips, Abbott Diabetes Care, Doncaster, VIC, Australia) from a drop of blood obtained from a tail nick. Within each of the four experimental groups, rats were randomly selected to undergo one of 3 hypoglycemia protocols. Protocol 1: recurrent hypoglycemia (“3×-hypo”; Non-D Chow *n* = 5, Non-D Keto *n* = 5, STZ-D Chow *n* = 4, STZ-D Keto *n* = 5); 5 U/kg of insulin (human recombinant, I2643, Sigma-Aldrich, Macquarie Park, NSW, Australia) was injected intraperitoneally (i.p.) for 3 consecutive days. Protocol 2: single hypoglycemia (“1×-Hypo”; Non-D Chow *n* = 5, Non-D Keto *n* = 7, STZ-D Chow *n* = 5, STZ-D Keto *n* = 5); vehicle (normal saline) was injected i.p. on days 1 and 2; insulin (5 U/kg) was injected on day 3. Protocol 3: no hypoglycemia (“No Hypo”; Non-D Chow *n* = 5, Non-D Keto *n* = 5, STZ-D Chow *n* = 5, STZ-D Keto *n* = 5); normal saline was injected i.p. on three consecutive days. All the injections were done at 9:00 am and food was removed from the cages and returned 2 h after injections on days 1 and 2; water was provided ad libitum. Blood BG and BHB were measured at baseline and 2 h post insulin/vehicle injection. 

Two hours after insulin administration on day 3 of each experimental protocol and following the last BG and BHB measurements, animals were deeply anaesthetised with sodium pentobarbital (~1 mL of 65 mg/mL injected i.p., LETH, Lethabarb, Virbac, Milperra, NSW, Australia). Blood was collected through cardiac puncture into EDTA Vacutainer tubes (BD367525, BD Vacutainer K2 EDTA, Becton Dickinson, Macquarie Park, NSW, Australia) and plasma was separated by centrifugation (4750 rpm, 10 min, 4 °C), aliquoted and stored at −80 °C. Rats were then transcardially perfused with 400 mL PBS followed by 400 mL of 4% PFA. The brain and the pancreas were dissected and post-fixed in 4% PFA for 24 h. After, the brain was transferred into PBSm (PBS with 0.1% Merthiolate (Thimerosal, T5125, Sigma-Aldrich, Macquarie Park, NSW, Australia)) for storage at 4 °C. Pancreas was stored in 70% ethanol at room temperature.

### 2.4. Epinephrine, C-Peptide, Insulin, Glucagon ELISAs

The following ELISA kits were used for the determination of: Epinephrine (KA3837, Abnova, Taipei, Taiwan), C-peptide (90055, Crystal Chem, IL, USA), insulin (90010, Crystal Chem, IL, USA) and glucagon (10-1281-01, with Technical Note No: 34-162, Mercodia, Uppsala, Sweden) following manufacturer’s instructions. All samples were run in duplicates. Insulin was measured only in groups where exogenous insulin was not injected (“No Hypo”). C-peptide was measured only in diabetic “No Hypo” groups (STZ-Chow and STZ-Keto).

### 2.5. Immunohistochemical Analysis of Lateral and Perifornical Hypothalamus

PFA-fixed brains were sectioned coronally on a vibrating microtome (Vibratome VT1200S, Leica Microsystems, Macquarie Park, NSW, Australia) at 40 µm in 1:5 series. Sections containing OX-positive cell populations in the lateral and perifornical hypothalamus were collected between −2.40 mm and −3.12 mm from Bregma [21] and subjected to a free-floating immunostaining protocol, as described in detail before [22]. OX-containing neurons were detected with guinea pig polyclonal anti-Orexin A/B antibody (#389 104, Synaptic Systems GmbH, Goettingen, Germany) at 1:1000 dilution and c-Fos was detected with rabbit polyclonal anti-c-Fos antibody (ab190289, Abcam, Cambridge, UK), 1:1000 dilution. Secondary antibodies were donkey anti-guinea pig AlexaFluor488, 1:500 (706-546-148, Jackson Immunoresearch, West Grove, PA, USA) and donkey anti-rabbit Cy5, 1:500 (711-175-152, Jackson Immunoreserach, West Grove, PA, USA), respectively. A Zeiss Axio Imager Z2 microscope (Zeiss, Germany) with 10× objective was used to capture immunofluorescent staining. OX-positive, c-Fos-positive, and dually labelled OX/c-Fos-positive cells in the lateral and perifornical hypothalamic areas (4–6 sections per animal) were counted bilaterally by the operator blinded to experimental conditions. For group comparisons, the counts were expressed as the percentage of activated (c-Fos-positive) OX-positive neurons of the total OX-positive population.

### 2.6. IHC for Insulin and Glucagon (Pancreas)

The splenic lobe of the pancreases from all STZ-diabetic rats, 5 Non-D Chow and 5 Non-D Keto rats were dissected, paraffin embedded and sectioned at 5 µm on a microtome (Ergostar Microm HM200, Microm, Walldorf, Germany). The sections were mounted on glass slides and stained for insulin (1:100, #4590S rabbit polyclonal anti-insulin antibody, Cell Signalling Technology, Danvers, MA, USA) and glucagon (1:2000, #G2654 mouse monoclonal anti-glucagon antibody, Sigma-Aldrich, Macquarie Park, NSW, Australia). The corresponding fluorophore-conjugated secondary antibodies were donkey anti-rabbit AlexaFluor488 (1:500, 711-546-152, Jackson Immunoresearch, West Grove, PA, USA) and donkey anti-mouse Cy5 (1:500, 715-175-151, Jackson Immunoresearch, West Grove, PA, USA), respectively. Pancreatic islets (8–12 per animal) were visualised with Zeiss Axio Imager Z2 fluorescence microscope (Zeiss, Germany) under 10× objective. The ratio of insulin-positive to glucagon-positive staining within each islet was determined in FIJI [23] by measuring the thresholded area of each channel by an operator blinded to experimental conditions. 

### 2.7. Statistical Analysis

Data were analysed in Graphpad Prism 9 statistical software. Unless stated otherwise, data are given as means and SEMs, with statistical significance set at *p* < 0.05. Comparisons of means between ≥3 groups were assessed by one-way ANOVA with Holm-Šídák multiple comparisons test. The differences between 2 groups were evaluated with Mann–Whitney test or Welch’s *t*-test. Correlations were analyzed using Spearman’s correlation. Analysis was performed on log-transformed data for epinephrine and glucagon concentrations due to the heteroscedastic nature of the untransformed data; the values reported in the text are untransformed concentrations (pg/mL) and 95% confidence intervals.

## 3. Results

### 3.1. Ketogenic Diet Differentially Affects Weight, Blood Glucose (BG) and β-Hydroxybutyrate (BHB) of Healthy and STZ-Diabetic Rats

**Figure 1:**
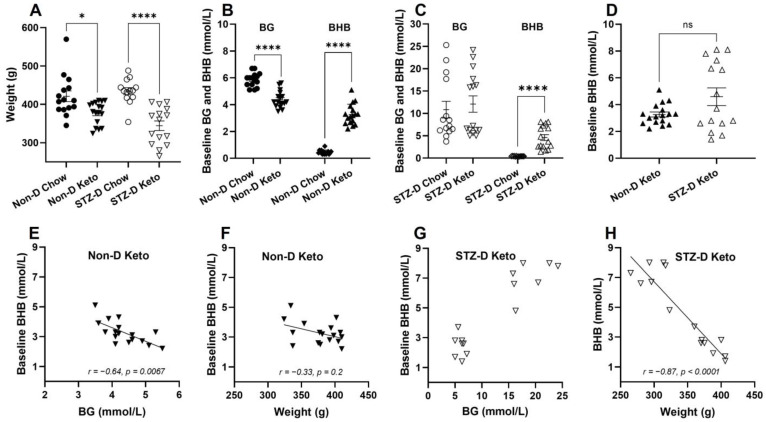
The effects of three weeks of nutritional ketosis of bodyweight, blood glucose (BG) and β-hydroxybutyrate (BHB) of non-diabetic and STZ-diabetic rats. Diabetes was induced with a single i.p. injection of 60 mg/kg STZ (citrate buffer was used in non-diabetic controls), followed by 3 weeks of dietary intervention. (**A**) Significantly lower bodyweight in ketogenic diet-fed non-diabetic (Non-D Keto) and diabetic (STZ-D Keto) rats compared to respective chow-fed controls; (**B**) Non-D Keto rats had lower BG and higher BHB concentrations than chow fed controls; (**C**) Keto diet did not affect BG in STZ-diabetic rats, but significantly elevated BHB; (**D**) No differences in baseline BHB of Non-D Keto and STZ-D Keto rats were observed; (**E**) Baseline BHB inversely correlated with BG in Non-D Keto rats; (**F**) BHB did not significantly correlate with weight in Non-D Keto rats; (**G**) Highest BHB concentrations in STZ-D Keto rats corresponded to the highest BG; (**H**) A strong inverse correlation between bodyweight and BHB was detected in STZ-D Keto rats. Data were analysed by Mann–Whitney tests (**A**–**C**), Welch’s *t*-test (**D**), Spearman’s correlation (**E**,**F**). Data shown as mean ± SEM, * *p* < 0.05, **** *p* < 0.0001.

The effects of nutritional ketosis on body weight, blood glucose (BG) and β-hydroxybutyrate (BHB) were assessed after three weeks of keto-diet (Keto) or normal chow (Chow) feeding in non-diabetic (Non-D) and STZ-diabetic (STZ-D) rats. Regardless of the diabetes status, keto-diet fed rats had significantly lower body weight (Figure 1A, 377 ± 7.1 (Non-D Keto, *n* = 17) vs. 421 ± 13.9 (Non-D Chow, *n* = 15) g, *p* < 0.05; 344 ± 12.5 (STZ-D Keto, *n* = 15) vs. 434 ± 9.1 (STZ-D Chow, *n* = 13) g, *p* < 0.0001). Blood glucose concentration was also lower in keto-diet fed rats (Figure 1B, 5.9 ± 0.13 (Non-D Chow) vs. 4.4 ± 0.15 (Non-D Keto) mmol/L, *p* < 0.0001), but only in the non-diabetic animals (Figure 1C). As expected, three weeks of ketogenic diet feeding significantly increased blood BHB in non-diabetic (Figure 1B, 3.3 ± 0.18 mmol/L) and STZ-diabetic animals (Figure 1C, 4.6 ± 0.66 mmol/L), compared to chow-fed controls (Figure 1B, 0.5 ± 0.04 mmol/L, *p* < 0.0001, Figure 1C, 0.4 ± 0.02 mmol/L). No significant differences in BHB concentration were detected between non-diabetic and diabetic animals, although the range of measured concentrations was wider in the STZ-diabetic group (Figure 1D). Despite the similar level of diet-induced ketosis in non-diabetic and diabetic animals, the correlations between BHB, BG and bodyweight appeared to be opposite—under non-diabetic conditions, higher ketosis was associated with lower BG (Figure 1E, r = −0.64, *p* = 0.0067, *n* = 17) and did not correlate with bodyweight, whereas in the diabetic state animals with highest BG also had the highest BHB (Figure 1G), and these higher levels of BHB had a negative correlation with bodyweight (Figure 1H, r = −0.87, *p* < 0.0001, *n* = 15).

### 3.2. STZ-Diabetes Model

**Figure 2:**
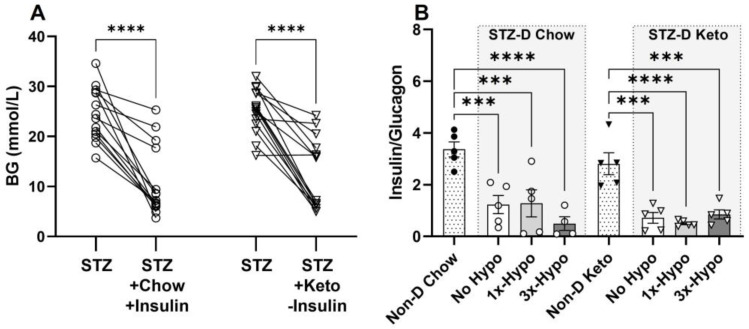
Streptozotocin (STZ) induces hyperglycemia and pancreatic damage in chow and keto-fed rats. (**A**) A single 60 mg/kg dose of STZ, administered i.p. significantly increased baseline BG levels in both Chow and Keto-fed rats; insulin supplementation decreased BG in the STZ-D Chow rats to the same level as ketogenic diet alone, without insulin supplementation, in STZ-D Keto group; (**B**) The ratio of insulin (β-cells)/glucagon (α-cells) producing cells in pancreatic islets of all STZ-D groups (No Hypo, 1×-Hypo and 3×-Hypo) of chow-fed and keto-fed rats was significantly reduced compared to vehicle (citrate) controls (Non-D Chow and Non-D Keto, respectively). Data were analysed by one-way ANOVA with Holm-Šídák multiple comparisons test and presented as mean ± SEM, *** *p* < 0.001, **** *p* < 0.0001.

**Figure 3:**
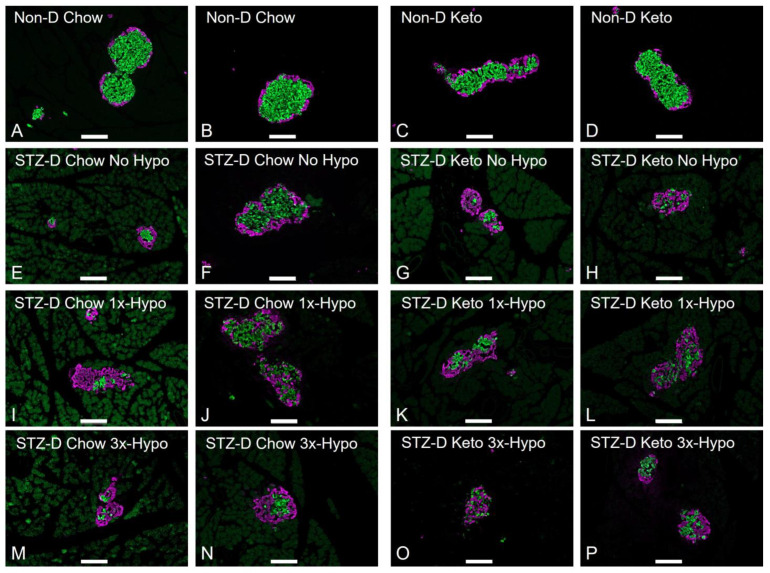
Changes in pancreatic islet morphology in STZ-injected rats. 5-µm sections of the pancreas were stained for insulin (green) and glucagon (purple); representative images are shown (scale bar 100 µm). (**A**,**B**) Non-diabetic chow-fed control; (**C**,**D**) Non-diabetic keto-fed control; (**E**,**F**) STZ-treated Chow No Hypo control; (**G**,**H**) STZ-treated Keto No Hypo Control; (**I**,**J**) STZ-treated Chow single hypoglycemia; (**K**,**L**) STZ-treated Keto single hypoglycemia; (**M**,**N**) STZ-diabetic Chow recurrent hypoglycemia; (**O**,**P**) STZ-diabetic Keto recurrent hypoglycemia.

In order to maximise the relevance of our rat model of type 1 diabetes to the human condition, STZ-D chow-fed animals were supplemented with slow-release insulin implants for the duration of the study; this treatment did not normalise BG, but significantly reduced STZ-induced hyperglycemia (Figure 2A, 24.5 ± 1.4 mmol/L pre-implant vs. 10.8 ± 1.9 mmol/L one week post-implant, *p* < 0.0001). Keto-diet STZ-D groups were not supplemented with insulin due to profound hypoglycemia observed with insulin treatment (pilot experiments, Supplementary Figure S1). However, having established a positive glucose-lowering effect of the ketogenic diet in healthy rats, we investigated whether ketogenic diet consumption, in the absence of insulin supplementation, would improve STZ-induced hyperglycemia. The diet alone reduced glycemia to the levels measured in STZ-D Chow-insulin supplemented animals (Figure 2A, 25.2 ± 1.1 mmol/L pre-diet vs. 12.1 ± 1.8 mmol/L one week post-diet, *p* < 0.0001). To verify the STZ-induced damage at the conclusion of the study, we analysed 5µm formalin-fixed paraffin-embedded sections of the pancreas immunolabeled for insulin (β-cells) and glucagon (α-cells). Immunohistochemical analysis of pancreases from STZ-treated animals showed significantly damaged islets of Langerhans in all animals, as assessed by measuring the ratio of insulin- to glucagon-immunopositive cells within the islets and comparing them to islets from non-diabetic rats (Figure 2B, Non-D Chow vs. STZ-D Chow, *p* < 0.0001; Non-D Keto vs. STZ-D Keto, *p* < 0.001–0.0001). STZ treatment led to a significant loss of β-cells, hyperplasia of α-cells and loss of islet integrity (Figure 3 shows representative examples from each group). Chow-fed STZ-treated rats, supplemented with insulin exhibited a wide range of pancreatic damage severity, with one animal being within the normal range (Figure 2B, STZ-D Chow 1×-Hypo group, Figure 3E,F,I,J,M,N). This suggests that insulin supplementation may lead to some regeneration of β-cells, which is in agreement with previous reports [24]. In contrast, all STZ-D Keto-fed rats had very similar, high degree of β-cell damage, clearly delineated from what was observed in the normal, non-diabetic animals (Figure 2 and Figure 3G,H,K,L,O,P). Overall, these results indicate that STZ treatment was successful and the normoglycemic BG values can be attributed to either insulin supplementation or keto-diet intervention.

### 3.3. Hypoglycemia-Associated Autonomic Failure (HAAF) Model and Counterregulatory Hormone Response—Non-Diabetic and Diabetic Conditions

To establish a model of hypoglycemia-associated autonomic failure (HAAF), we used a previously reported insulin injection protocol [25]. All animals that were injected with 5 U/kg of insulin i.p. (1×-Hypo—a single injection, 3×-Hypo—once-daily injections over 3 days) reached the hypoglycemic threshold of BG ≤ 3.9 mmol/L within 2 h of injections, regardless of the diabetic state. Control groups (No Hypo), injected with saline, stayed normoglycemic. In Keto-fed animals, BHB concentration also decreased with insulin injections. However, in recurrent hypoglycemia groups (3×-Hypo) the decreases in BHB were more variable than the decreases in BG in the same groups. Supplementary Table S1 provides complete data for BG and BHB changes in each experimental group.

**Figure 4:**
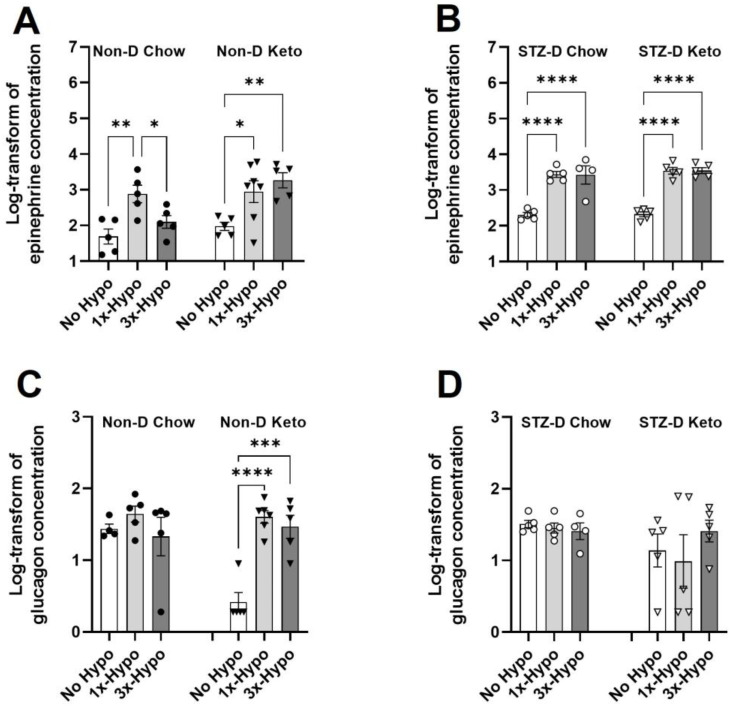
Ketogenic diet differentially affects hypoglycemia counterregulatory hormones in non-diabetic and diabetic rats. (**A**) Recurrent hypoglycemia (3×-Hypo) reduces epinephrine counterregulatory response in non-diabetic chow-fed rats; no reduction is observed in non-diabetic keto-fed rats; (**B**) Epinephrine counterregulatory response to recurrent hypoglycemia (3×-Hypo) is preserved in STZ-diabetic chow- and keto-fed rats; (**C**) Single (1×-Hypo) or recurrent (3×-Hypo) hypoglycemia did not induce glucagon counterregulatory response in non-diabetic chow-fed animals, but significantly increase its release in non-diabetic keto-fed rats; (**D**) No changes in glucagon concentration were detected in STZ-diabetic animals. Log-transformed concentrations of epinephrine and glucagon (pg/mL) were analysed by one-way ANOVA with Holm-Šídák multiple comparisons test. Data are mean ± SEM, * *p* < 0.05, ** *p* < 0.01, *** *p* < 0.001, **** *p* < 0.0001.

Having established that hypoglycemic threshold can be achieved in all 1×-Hypo and 3×-Hypo groups under non-diabetic and diabetic conditions in both keto- and chow-fed groups of animals, we proceeded to assess the impairment of neuroendocrine counterregulatory response. A diminished epinephrine response after recurrent hypoglycemia is considered a hallmark of a successfully established model of HAAF (reviewed in Sankar et al. [26]). Consistent with previous reports, our study found that recurrent injections of insulin in Non-D Chow group resulted in a significant attenuation of epinephrine release (Figure 4A, 170.7 pg/mL (95% CI: −4.1–345.5), *n* = 5) compared to 1×-Hypo group (Figure 4A, 1317 pg/mL (95% CI: −464.7–3099), *p* < 0.05, *n* = 5). In contrast, Non-D Keto animals were protected from the diminution of epinephrine release in the recurrent hypoglycemia (3×-Hypo) group (Figure 4A, 2181 pg/mL (95% CI: 29.2–4333) 1×-Hypo, *n* = 7 vs. 2691 pg/mL (95% CI: 155.4–5226) 3×-Hypo, *n* = 5, *p* > 0.05). In STZ-D groups, epinephrine response to both single and repeat hypoglycemia was strong and there was no attenuation in epinephrine response to recurrent hypoglycemia in either diet group (Figure 4B, STZ-D Chow 1×-Hypo 2958 pg/mL (95% CI: 1238–4678) *n* = 5 vs. 3×-Hypo 3804 pg/mL (95% CI: −491.8–8099) *n* = 4, *p* > 0.05; STZ-D Keto 1×-Hypo 3790 pg/mL (95% CI: 1518–6061) *n* = 5 vs. 3×-Hypo 3769 pg/mL (1806–5732) *n* = 5, *p* > 0.05).

Unlike epinephrine, glucagon is a less reliable marker of counterregulatory response impairment, especially in people with type 1 diabetes [27,28], and highly variable results are reported in animal HAAF models [29]. In our study we found there were no significant differences in glucagon concentration in any of the Chow-fed groups (Figure 4C,D, Non-D and STZ-D) or STZ-D Keto group (Figure 4D), suggesting that glucagon release is unaffected by hypoglycemia in these groups. In Non-D Keto rats baseline (No Hypo) glucagon concentration was very low (Figure 4C, 3.3 pg/mL (95% CI: −0.6–7.2), and was significantly increased in both single (Figure 4C, 1×-Hypo 44 pg/mL (95% CI: 23.6–64.4) and recurrent (Figure 4C, 3×-Hypo 36.8 pg/mL (95% CI: 7.2–66.3) hypoglycemia groups.

**Figure 5:**
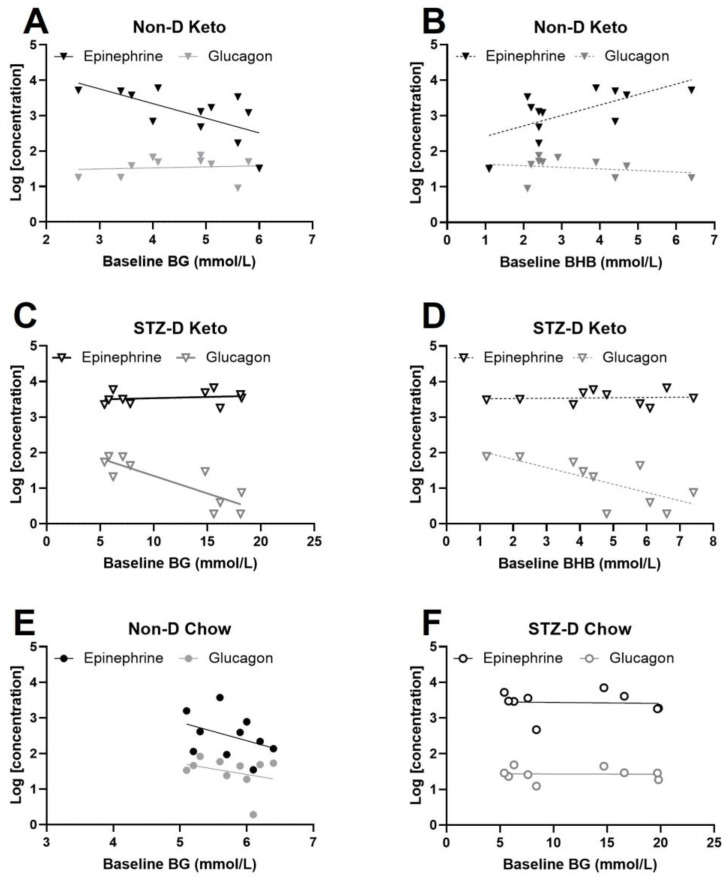
Baseline BG and BHB correlate with post-hypoglycemic epinephrine. Plasma epinephrine and glucagon concentrations were measured in animals subjected to a single (1×-Hypo) or recurrent (3×-Hypo) insulin injections; in the 3×-Hypo groups epinephrine and glucagon were measured after the final insulin injection. (**A**) In non-diabetic keto-fed rats baseline BG inversely correlated with plasma epinephrine, but not glucagon, measured 2 h after insulin injection (*n* = 12); (**B**) Direct correlation was detected between baseline BHB and epinephrine in non-diabetic keto-fed rats, and no correlation with glucagon; (**C**) Baseline BG did not correlate with epinephrine or glucagon in STZ-diabetic keto-fed rats (*n* = 10); (**D**) Baseline BHB significantly correlated with glucagon, but not epinephrine in STZ-diabetic keto-fed rats; (**E**) Baseline BG was not associated with changes in epinephrine or glucagon in non-diabetic chow-fed rats (*n* = 10) or (**F**) STZ-diabetic chow-fed rats (*n* = 9). Concentrations of epinephrine ang glucagon (pg/mL) were log-transformed for analysis. Data analysed by Spearman’s correlation. Datapoints are individual animals.

Given the existing reciprocal relationship between BG and BHB (Figure 1) and the opposite direction of this correlation in non-diabetic and STZ-diabetic keto-fed animals, we investigated how baseline levels of BG and BHB are related to changes in counterregulatory hormones following insulin administration. The correlation between baseline BG after insulin injection (1×-Hypo and day 3 values of 3×-Hypo groups) and final epinephrine or glucagon concentrations was tested with Spearman correlation. We found epinephrine concentration in non-diabetic, keto-diet fed rats changed in proportion to baseline BG after insulin injection: higher baseline BG was associated with lower epinephrine concentration (Figure 5A, Spearman’s r = −0.67, *p* = 0.018). Baseline BHB also significantly correlated with epinephrine post-insulin, but in the direction opposite to BG—lower baseline BHB corresponded to lower epinephrine concentrations (Figure 5B, Spearman’s r = 0.57, *p* = 0.027); this was not surprising given the inverse correlation between BG and BHB in non-diabetic keto-fed rats (Figure 1E). Taken together, these findings suggest that the magnitude of epinephrine counterregulatory response varies in proportion to starting BG and BHB (baseline). In contrast, no correlations were observed between post-insulin epinephrine and baseline BG/BHB in STZ-diabetic keto-fed rats (Figure 5C,D).

Since BHB and BG in STZ-D Keto-fed animals showed a positive association (Figure 1G), the animals with the highest values for both (hyperglycemic and hyperketonemic) were expected to have the highest degree of pancreatic damage induced by STZ, and thus have the lowest concentrations of glucagon and insulin (insulin was not measured in 1x-Hypo and 3×-Hypo groups due to methodological constraints, but significantly lower insulin concentration was measured in the No Hypo group (Supplementary Figure S2). Indeed, glucagon concentration negatively correlated with baseline BHB (Figure 5D, Spearman’s r = 0.8, *p* = 0.0075). There appeared to be a similar correlation with BG (Figure 5C), but the values were clustered in two groups (normoglycemic and hyperglycemic), rather than being homogenously distributed within the range of assessed concentrations, making the calculations of correlation coefficient biased and therefore not reported. Nevertheless, BHB concentrations in STZ-D Keto-fed rats appear to reflect the extent of STZ-induced α-cell dysfunction. No correlations between BG and epinephrine or glucagon were detected in chow-fed animals (Figure 5E,F).

### 3.4. Hypothalamic Orexin Neurons Activity Is Attenuated by Recurrent Hypoglycemia in STZ-D Keto Rats

**Figure 6:**
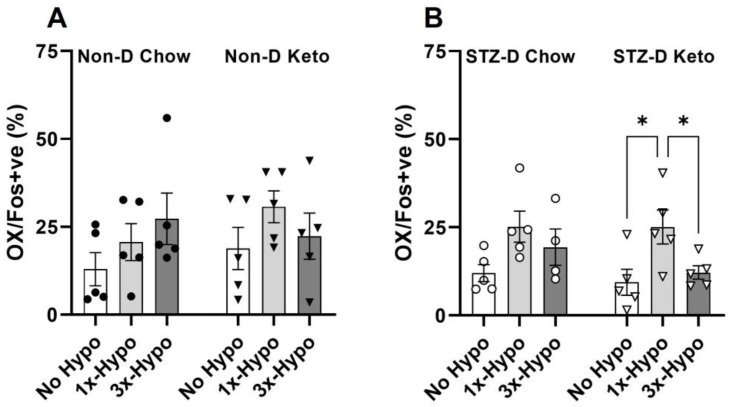
Recurrent hypoglycemia attenuates OX neurons activation in STZ-diabetic keto-fed rats. (**A**) The proportion of Fos-positive OX neurons did not change with hypoglycemic challenge in any of the non-diabetic groups of rats; (**B**) Single (1×-Hypo) hypoglycemia increased the proportion of activated OX neurons in STZ-diabetic keto-fed rats compared to the No Hypo control and recurrent hypoglycemia (3× Hypo) attenuated this increase; no differences in OX activity were detected in STZ-diabetic chow-fed rats. Data analysed by one way ANOVA with Holm–Šídák multiple comparisons test. Data are mean ± SEM, * *p* < 0.05.

In contrast to previous studies, we did not observe any changes in Fos protein expression in hypothalamic OX neurons in non-diabetic chow or keto-fed animals following a single or recurrent hypoglycemia (Figure 6A). On the other hand, STZ-D Keto-fed animals showed a significant increase in OX activity following a single hypoglycemia and attenuation of this response in the recurrent hypoglycemia group (Figure 6B, STZ-D No Hypo 9.4 ± 3.7% vs. 1×-Hypo 25.1 ± 4.8%, *p* < 0.05, vs. 3×-Hypo 12.2 ± 1.9%, *p* < 0.05). This is in discordance with epinephrine release observed in 3×-Hypo group, which showed no diminution by an antecedent hypoglycemia, suggesting that attenuation of OX activation is not translated to the downstream sympathoadrenal response, and perhaps related to other physiological functions modulated by the OX neurons, such as arousal, exploration, and/or food seeking behaviour. No significant increase in OX Fos expression was evident in 1×-Hypo or 3×-Hypo groups of STZ-D Chow-fed rats (Figure 6B).

## 4. Discussion

Here, we characterised the neurohormonal and metabolic changes associated with nutritional ketosis in non-diabetic and STZ-diabetic rat models of hypoglycemia-associated autonomic failure (HAAF). We demonstrated that epinephrine and glucagon counterregulatory responses are not attenuated by antecedent hypoglycemia under the conditions of diet-induced ketosis. In contrast, the activity of energy-sensing and wakefulness promoting hypothalamic OX neurons in the STZ-diabetic keto-fed rats was significantly reduced by recurrent hypoglycemia. Additionally, our study identified a significant correlation between the magnitude of the neuroendocrine response to hypoglycemia and baseline concentrations of both BHB and BG. Overall, our study suggests that in insulin-deficient diabetes, diet-induced ketosis might attenuate the responsiveness of the hypothalamic OX neurons to recurrent hypoglycemia by mechanisms that most likely do not involve and/or do not affect the sympathoadrenal catecholamine response. 

We recently reported that in anaesthetised, keto-diet fed, non-diabetic rats the magnitude of the epinephrine counterregulatory response to a single episode of insulin-induced hypoglycemia, assessed by measuring plasma epinephrine concentration 2 h after insulin injection, is large but triggered at substantially lower BG levels compared to chow-fed animals [17]. Here, we first sought to determine whether this delayed, yet robust, neuroendocrine response is also evident in a more physiologically relevant conscious animal model and if it is attenuated by repeated hypoglycemia exposure. We found that plasma epinephrine concentration, measured 2 h after a single insulin injection in non-diabetic rats, was similar in keto diet and chow-fed animals. In striking contrast to the chow-fed rats, however, recurrent hypoglycemia did not diminish this response in keto-fed rats, suggesting a preservation of sympathoadrenal counterregulation [25].

Consistent with previous findings in animals and humans [20,30,31], we also report a significant glucose-lowering effect of nutritional ketosis. Interestingly, the lower the initial BG concentration was, measured just prior to insulin injection, the larger was the magnitude of epinephrine counterregulatory response (Figure 5). Unlike many animal models of HAAF (reviewed in [26]), our model comprised of animals not fasted prior to insulin injection, which resulted in a wider range of baseline BG values, particularly in the keto-fed group (2.6–6.1 mmol/L). This spread allowed us to detect an inverse correlation between starting BG and epinephrine counterregulatory response. Previous hypoglycemic clamp studies in humans showed that the epinephrine counterregulatory response is triggered by a surprisingly wide range (2.7–4.1 mmol/L) of BG concentrations [32] and variations in the magnitude of the response is determined by the absolute hypoglycemic BG values at which the response occurred; this is hypothesised to explain the variability of hypoglycemia symptoms among studied individuals. Our findings are complementary to this, and it seems plausible that the animals with relatively low BG at the time of insulin injection reached the hypoglycemic threshold for epinephrine faster, or might have already been releasing epinephrine and potentially had more circulating epinephrine 2 h post insulin injection when it was quantified. 

The observed reciprocal relationship between BG and BHB in keto-diet fed rats, with BG inversely correlating with BHB under non-diabetic conditions, is in line with previous studies on fasting-induced ketogenesis [33,34]. In addition to fasting, a similar metabolic switch to fat oxidation is also induced by carbohydrate restricted high fat ketogenic diets [34], and therefore it is unsurprising to detect elevated circulating ketone bodies concurrently with decreased BG concentration. By the same logic, the positive correlation between BHB and epinephrine opposes the correlation between BG and epinephrine. Together, these two metrics can potentially predict the magnitude of epinephrine response and symptoms associated with hypoglycemia; this warrants further investigation.

In the current study we attempted to emulate the features of human type 1 diabetes by supplementing the chow-fed STZ-diabetic rats with slow-release insulin implants. Insulin supplementation improved BG in all animals, and the most normoglycemic animals had the highest β/α-cell ratio, a sign of probable β-cell regeneration [24]. Insulin supplementation of STZ-diabetic, keto diet-fed animals proved to be unfeasible due to severe hypoglycemia experienced by all animals (Supplementary Figure S1). Keto-diet fed rats, without insulin supplementation showed a significant decrease in BG after three weeks. Interestingly, both the magnitude and variability of this decrease was equivalent to that achieved in the STZ-chow-fed rats supplemented with insulin. Previous reports by Al-Khalifa et al. showed a complete normalisation of BG in STZ-diabetic rats fed a ketogenic diet for 4 weeks [35], however in that study the animals were sustained on the ketogenic diet for 8 weeks prior to the induction of diabetes. In a separate study, when STZ-diabetes was induced concurrently with initiation of the ketogenic diet feeding [36], BG decreased, but did not normalise and showed a wide range of BG concentrations among animals, similar to our study. These findings suggest that a ketogenic diet regimen may be effective in normalising BG in diabetic animals without additional insulin treatment, at least for a short period of time. 

Ketogenic diet consumption leads to weight loss in overweight and obese humans [37] and reduced weight gain in normal weight rodents [38,39], largely due to decreased food intake [40]. Consistent with the literature, our study found that both non-diabetic and STZ-diabetic keto-fed rats had lower bodyweights than the respective chow control animals. Only in STZ-diabetic keto-fed rats there was a strong negative correlation between BHB and weight and the animals with the highest blood BHB also had the highest BG, which is opposite to what was observed in the non-diabetic keto-fed rats. Whereas lower BG and elevated BHB (within the reference range) is considered a normal physiological response to the high fat/low carbohydrate diet consumption, as discussed above, hyperketonemia and hyperglycemia together with weight loss, hypoinsulinemia and hyperglucagonemia may indicate a catabolic state which can occur in poorly controlled or newly diagnosed diabetes [41]. 

Before the discovery of insulin, very low carbohydrate ketogenic diets, along with extended fasting, were non-pharmacological strategies to manage type 1 diabetes. This approach afforded some improvements in quality of life to people with type 1 diabetes as the amount of endogenously produced insulin required to metabolise very limited quantities of dietary carbohydrates, was also significantly reduced [6]. As the disease develops, however, progressive destruction of β-cells leads to absolute insulin deficiency, hyperglycemia and unrestricted pathological increase in fatty acid oxidation and ketogenesis. In our study we show that ketogenic diet feeding of STZ-diabetic rats resulted in the development of both disease stages—(1) initial insulin deficits are compensated by carbohydrate restriction: energy needs are met with increased fatty acid oxidation and ketone body metabolism; and (2) profound insulin deficiency with the development of hyperglycemia and hyperketonemia. Interestingly, this division occurred even though all the STZ-diabetic keto-fed rats had similar levels of pancreatic islet damage, as evidenced by immunohistochemical analysis (Figure 2B). It is possible that in our study the residual amount of insulin in normoglycemic STZ-diabetic animals was sufficient to restrict excessive ketogenesis (as insulin is a potent suppressor of ketogenesis [42]), enhance peripheral ketone uptake [43] and sustain it within nutritional ketosis range, whereas animals with presumed absolute insulin deficiency were hyperglycemic (although to a lesser degree than before the commencement of the diet), highly ketonemic and had the lowest levels of C-peptide (Supplementary Figure S3). This dichotomy in responses to diet induced ketosis in diabetic animals highlights the fine line between physiological ketosis which serves to decrease insulin requirements, alleviate hyperglycemia, and supplement the brain and other extrahepatic tissues with an additional energy source at the time of scarcity, such as hypoglycemia, and pathological ketonemia (ketoacidosis) associated with profound insulin deficiency and hyperglycemia. 

Because STZ-diabetic keto-fed rats displayed comparable baseline BG concentrations to the STZ-diabetic insulin-supplemented chow fed rats, both cohorts were subjected to the single or recurrent hypoglycemia protocols without further subdivision into hyperglycemic and normoglycemic groups. Here, we show that the epinephrine counterregulatory response to recurrent hypoglycemia was not reduced in STZ-diabetic rats, either chow-fed or keto-fed, and irrespective of whether ketosis was physiological or pathological. The results obtained for the STZ-diabetic chow-fed insulin supplemented animals agree with Inouye’s [44] findings which showed an intact epinephrine response in insulin treated STZ-diabetic rats, hypothesised to be due to the enhanced expression of adrenal enzymes essential for catecholamine synthesis. In this study, however, insulin supplementation did not prevent attenuation of the corticosterone counterregulatory response, suggesting differential effects of recurrent hypoglycemia on hypothalamo-pituitary axis function and sympathoadrenal counterregulation. 

Our previous study in anaesthetised rats showed a significant increase in adrenal sympathetic nerve activity, adrenal chromaffin cell activity, and epinephrine release during a single episode of hypoglycemia with a corresponding activation of C1 neurons in the rostral ventrolateral medulla (RVLM) [17,25] in both chow-fed and keto-fed rats. In addition to the epinephrine counterregulatory response, C1 neurons are essential for promoting a feeding response to glucoprivation and hypoglycemia, by acting on hypothalamic OX neurons [45]. At the same time, OX neurons play a role in mediating the sympathoadrenal response to hypoglycemia through the activation of orexin type 2 receptors in the RVLM [46] and stimulating adrenal epinephrine release. Besides the role in regulating the sympathetic outflow in response to hypoglycemia, OX neurons, through their diffuse projections [47], are implicated in the regulation of numerous physiological processes, including feeding, locomotion, sleep-wakefulness, reward-seeking behaviour, thermoregulation, and respiration [48,49,50,51]. Several reports have shown that OX neuron activation is modulated by antecedent hypoglycemia, although the findings are somewhat inconsistent [48,49,50,51]. Here, we found significant activation of OX neurons after a single hypoglycemia and attenuation of this activation by antecedent hypoglycemia in STZ-diabetic keto-fed rats, but not in any other group. 

As there was no blunting in the epinephrine response to recurrent hypoglycemia in STZ-diabetic rats, both chow-fed and keto-fed, the evidence for OX neurons adaptation to repetitive hypoglycemic stimulus in STZ-diabetic keto-fed rats is interesting and suggests a dissociation between the neuroendocrine counterregulation function and other functions of OX neurons, such as food seeking, locomotion and wakefulness. Although not investigated in the present study, it is plausible that following a single episode of hypoglycemia in STZ-diabetic keto-fed rats, the activated OX neurons, in addition to facilitating counterregulatory epinephrine release, also promoted vigilance and exploratory behaviour associated with food seeking to facilitate the restoration of organismal energy level. Such behaviour would be beneficial if an adequate, i.e., carbohydrate-rich, source of energy is found, however in our study, even when the food was returned 2 h post insulin injection on day 1 of the protocol (in the 3×-hypo group), it contained very little carbohydrates and therefore was not positively associated with rapid restoration of glucose homeostasis. It is possible that on the subsequent days of insulin injections in the 3×-hypo group, the reduced activation of OX neurons serves as an adaptation strategy aimed at energy conservation accompanied by diminished arousal and exploratory behaviour.

Furthermore, a recent study reported interesting new properties of OX neurons, with specific cell subpopulations responding differentially to reward-predicting cues, by either increasing their firing rate (activity) with increasing probability of the reward or increasing uncertainty of the reward [52]. If carbohydrate re-feeding can be considered a reward, the low probability (i.e., no carbohydrates on re-feeding, 0 probability) and decreased uncertainty (i.e., 100% certainty of not receiving the carbohydrates) of this reward may be associated with diminished OX activation on subsequent exposure to hypoglycemia (a reward-predicting cue in this case). Since the activation of OX neurons is strongly associated with waking during an insulin induced hypoglycemia [53], this may have serious repercussions during nocturnal hypoglycemia, when timely arousal is necessary to rectify the falling BG levels, even when the epinephrine response is unaffected and precedes the awakening [54]. Indeed, a recent preclinical study demonstrated that OX neurons are a promising therapeutic target for restoration of hypoglycemia awareness in the recurrent hypoglycemia settings [55].

## 5. Conclusions

In rodents with induced type 1 diabetes, the epinephrine counterregulatory response to recurrent hypoglycemia is not diminished under conditions of diet-induced moderate ketosis. However, the activity of hypothalamic OX neurons is attenuated, which can potentially lead to a higher arousal threshold during nocturnal hypoglycemia.

## Data Availability

Data is contained within the article or supplementary material.

## Supplementary Materials

The following supporting information can be downloaded at: https://www.mdpi.com/article/10.3390/metabo13010042/s1, Figure S1: Insulin supplementation in STZ-D Keto-fed rats induced profound hypoglycemia; Figure S2: Differential changes in circulating insulin and glucagon in non-diabetic and diabetic rats; Figure S3: C-peptide concentration inversely related to BG and BHB; Table S1: Blood glucose (BG) and β-hydroxybutyrate (BHB) before (0 min) and after (120 min) insulin (or vehicle) injection.
