# Supplementary material for: Still Excited, but Less Aroused—The Effects of Nutritional Ketosis on Epinephrine Response and Hypothalamic Orexin Neuron Activation Following Recurrent Hypoglycemia in Diabetic Rats
Source: Metabolites. 2022 Dec 27;13(1):42. doi: 10.3390/metabo13010042 (PMC9862750; doi:10.3390/metabo13010042)
Supplement: Supplementary file 1 [file metabolites-13-00042-s001.zip › metabolites-2112290-supplementary.pdf]

| | | Blood Glucose (mmol/L) | | | | | | Blood BHB (mmol/L) | | | | | |
|---|---|---|---|---|---|---|---|---|---|---|---|---|---|
| | | Day 1 | | Day 2 | | Day 3 | | Day 1 | | Day 2 | | Day 3 | |
| | | 0min | 120min | 0min | 120min | 0min | 120min | 0min | 120min | 0min | 120min | 0min | 120min |
| Non-D Chow | No Hypo (n=5) | 6.1±0.19 | 5.6±0.2 | 6.0±0.08 | 5.7±0.2 | 5.7±0.07 | 5.7±0.2 | 0.4±0.05 | 0.5±0.06 | 0.4±0.04 | 0.5±0.07 | 0.4±0.00 | 0.5±0.05 |
| | 1x-Hypo (n=5) | 5.8±0.21 | 5.8±0.3 | 5.4±0.16 | 5.8±0.14 | 5.7±0.23 | 1.6±0.18* | 0.5±0.11 | 0.5±0.05 | 0.5±0.05 | 0.6±0.06 | 0.4±0.04 | 0.4±0.03 |
| | 3x-Hypo (n=5) | 5.7±0.29 | 2.4±0.66* | 5.9±0.33 | 1.8±0.23* | 5.8±0.18 | 1.6±0.17* | 0.4±0.04 | 0.5±0.05 | 0.5±0.06 | 0.5±0.06 | 0.4±0.05 | 0.4±0.05 |
| | | | | | | | | | | | | | |
| Non-D Keto | No Hypo (n=5) | 4.5±0.37 | 5.0±0.28 | 5.0±0.25 | 5.3±0.17 | 5.0±0.34 | 5.1±0.22 | 3.5±0.44 | 2.5±0.34 | 2.7±0.29 | 2.1±0.19 | 2.2±0.35 | 2.3±0.54 |
| | 1x-Hypo (n=7) | 4.2±0.10 | 4.9±0.21 | 4.3±0.33 | 5.3±0.29 | 4.9±0.36 | 1.4±0.17* | 3.3±0.26 | 2.7±0.28 | 3.8±0.63 | 2.6±0.43 | 2.7±0.42 | 0.9±0.08 |
| | 3x-Hypo (n=5) | 4.9±0.34 | 1.5±0.20* | 4.2±0.32 | 2.1±0.38* | 4.1±0.52 | 1.9±0.16* | 2.9±0.29 | 1.1±0.2* | 4.0±0.8 | 2.8±1.02 | 4.0±0.79 | 1.5±0.15* |
| | | | | | | | | | | | | | |
| STZ-D Chow | No Hypo (n=5) | 9.1±2.26 | 6.8±2.08 | 9.1±2.98 | 7.2±1.82 | 10.3±2.36 | 7.7±2.09 | 0.4±0.02 | 0.4±0.04 | 0.4±0.03 | 0.4±0.03 | 0.4±0.05 | 0.4±0.03 |
| | 1x-Hypo (n=5) | 12.7±3.29 | 9.7±2.76 | 13.0±2.89 | 9.2±2.09 | 11.4±3.41 | 2.6±0.31* | 0.4±0.03 | 0.4±0.07 | 0.4±0.04 | 0.4±0.02 | 0.4±0.04 | 0.4±0.05 |
| | 3x-Hypo (n=4) | 10.7±4.93 | 2.6±0.27 | 11.9±3.01 | 2.3±0.48 | 11.8±2.25 | 2.2±0.24 | 0.4±0.05 | 0.3±0.06 | 0.3±0.03 | 0.3±0.05 | 0.3±0.04 | 0.4±0.12 |
| | | | | | | | | | | | | | |
| STZ-D Keto | No Hypo (n=5) | 10.1±2.41 | 9.8±2.36 | 10.7±2.86 | 10.3±1.91 | 11.2±1.97 | 10.8±1.85 | 3.8±1.30 | 3.9±1.53 | 4.3±1.41 | 3.3±1.09 | 3.2±1.11 | 3.6±1.43 |
| | 1x-Hypo (n=5) | 13.4±2.98 | 12.4±2.53 | 11.9±2.61 | 12.1±2.46 | 12.6±2.54 | 2.7±0.22* | 5.0±1.08 | 5.0±1.32 | 5.6±1.08 | 5.1±1.01 | 4.2±1.07 | 0.8±0.12* |
| | 3x-Hypo (n=5) | 12.8±4.34 | 2.9±0.68* | 9.9±2.18 | 2.1±0.19* | 10.5±2.54 | 2.2±0.13* | 5.0±1.22 | 2.4±0.74 | 4.9±1.15 | 2.0±0.79 | 5.1±0.67 | 1.4±0.22* |

**Table S1. Blood glucose (BG) and β-hydroxybutyrate (BHB) before (0min) and after (120min) insulin (or vehicle) injection.**

Data are mean ± SEM, *$p < 0.05$ vs 0min.

**Figure S1**

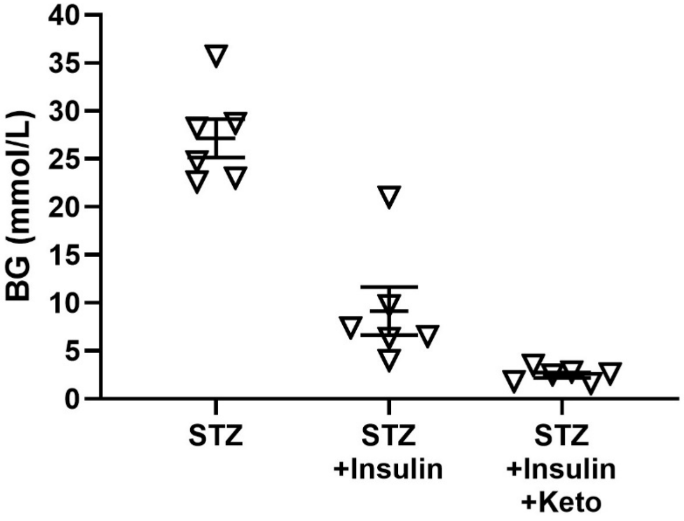


**Figure S1. Insulin supplementation in STZ-D Keto-fed rats induced profound hypoglycemia.** Following the induction of diabetes with STZ, rats (*n* = 6) were implanted with 1-2 continuous-release insulin pellets which reduced hyperglycemia within 48 h (STZ+Insulin). When subjected to a keto diet, all rats in the group became hypoglycemic within the first week of feeding (STZ+Insulin+Keto). The diet was replaced with the standard chow and animals were excluded from the study.

**Figure S2**

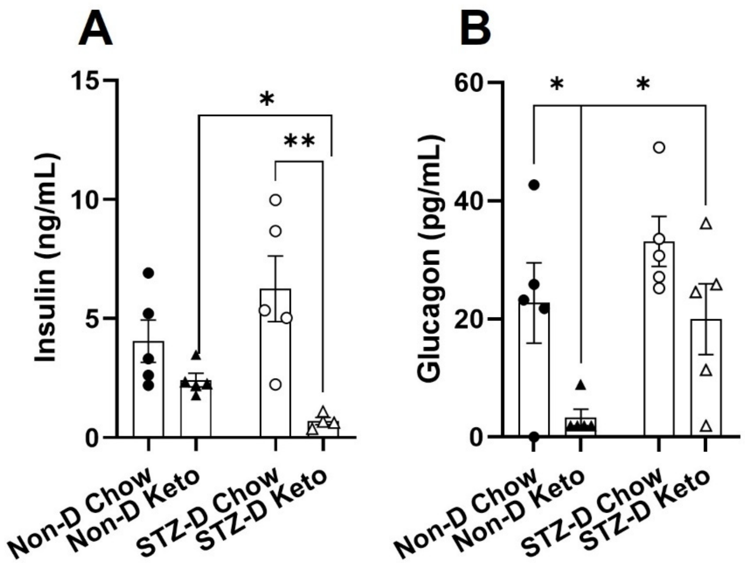


**Figure S2. Differential changes in circulating insulin and glucagon in non-diabetic and diabetic rats.** Plasma insulin and glucagon were measured in animals not subjected to hypoglycemia (No Hypo groups) to estimate the baseline levels under non-diabetic and diabetic conditions and assess the effects of diet-induced ketosis on both. A) STZ-D Keto-fed rats had significantly lower insulin than Non-D Keto-rats ($p < 0.05$) and STZ-D Chow-fed rats ($p < 0.01$); B) Baseline glucagon in Non-D Keto-fed rats was significantly lower than in Non-D Chow rats and STZ-D Keto rats ($p < 0.05$).

**Figure S3**

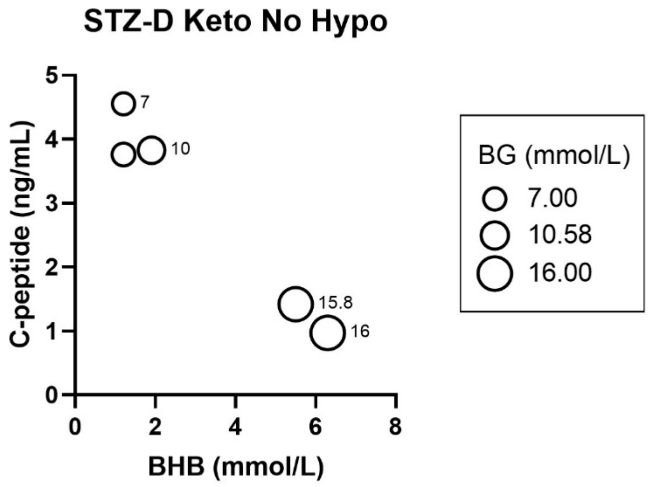


**Figure S3. C-peptide concentration inversely related to BG and BHB.** Plasma C-peptide was measured in STZ-D Keto-fed rats from the No Hypo group. Animals with highest BG and BHB had the lowest C-peptide concentration. The size of the circles indicates BG concentration and numbers show individual values.
